# Supplementary material for: End to end AI system for surgical gesture sequence recognition and clinical outcome prediction
Source: NPJ Digit Med. 2026 Jun 23;9:494. doi: 10.1038/s41746-026-02927-5 (PMC13319110; doi:10.1038/s41746-026-02927-5)
Supplement: Supplementary file 1 — Supplementary Information [file 41746_2026_2927_MOESM1_ESM.pdf]

Supplementary Information

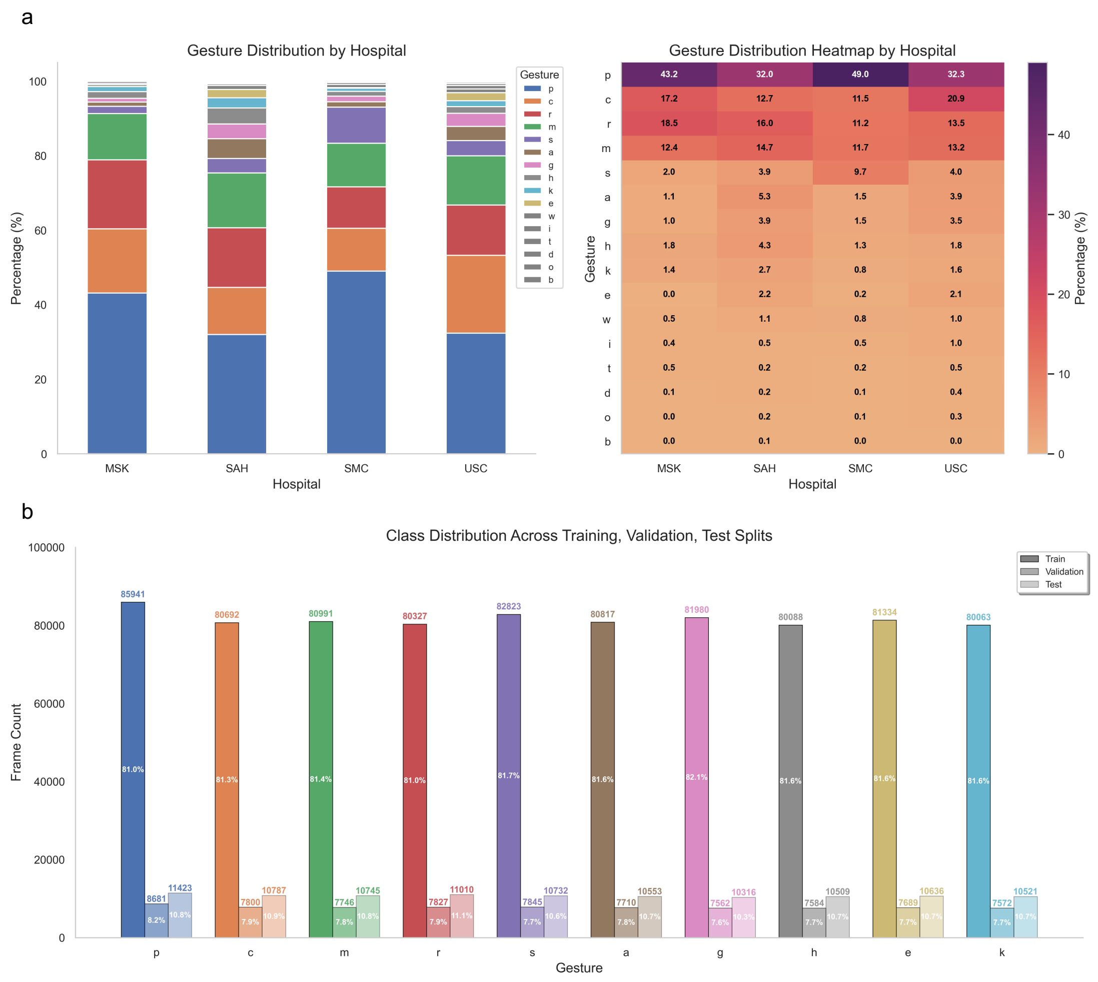

Supplementary Figure 1. Distribution of gestures across institutions and data splits. **a**, Gesture distribution across institutions, presented as stacked bar plots and heatmaps. **b**, Gesture class distributions across the training, validation, and test splits, with frame counts annotated.

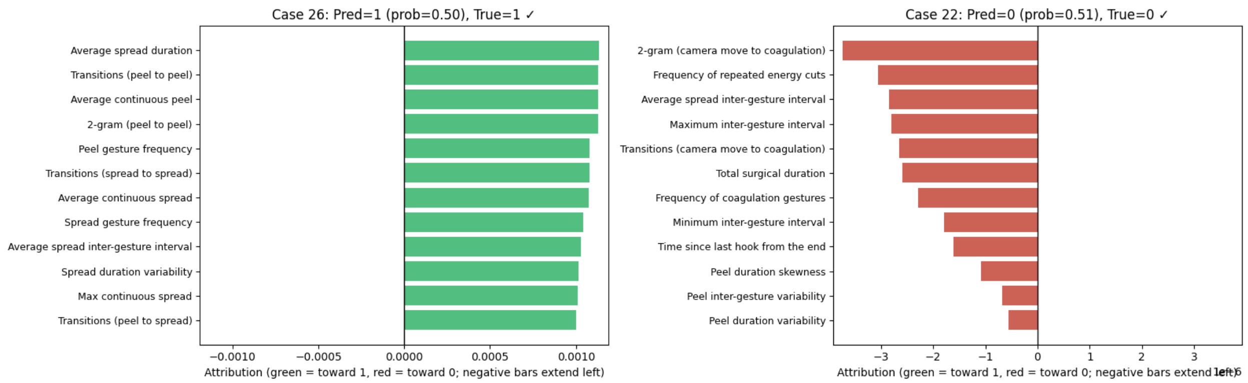

Supplementary Figure 2. Feature attributions for two representative cases.

Supplementary Table 1 Comparison of overlapping features between AI system and Ground Truth Based on Recovery of Erectile Function in 12 months (EF)

	F2O			Ground Truth		
Feature	Poor EF	Good EF	*p*-value	Poor EF	Good EF	*p*-value
dur_sum_p	142.020 ± 138.948	243.067 ± 240.817	0.0023	202.064 ± 181.159	292.157 ± 250.448	0.0187
avg_run_s	0.934 ± 0.640	1.274 ± 0.526	0.0031	1.181 ± 0.562	1.352 ± 0.496	0.0902
max_run_s	1.542 ± 1.704	2.476 ± 1.685	0.0035	2.375 ± 2.341	3.333 ± 2.238	0.0266
trans_p_s	0.0115 ± 0.0271	0.0293 ± 0.0427	0.0038	0.0197 ± 0.0229	0.0299 ± 0.0332	0.0398
trans_c_s	0.0037 ± 0.0143	0.0118 ± 0.0200	0.0075	0.0152 ± 0.0250	0.0240 ± 0.0308	0.0791
2gram_p_p	0.0636 ± 0.0602	0.0993 ± 0.1021	0.0113	0.1594 ± 0.0852	0.1993 ± 0.0842	0.0122
freq_g	0.1557 ± 0.1213	0.1047 ± 0.0771	0.0133	0.0419 ± 0.0375	0.0292 ± 0.0279	0.0499
freq_p	0.1451 ± 0.0924	0.1926 ± 0.1277	0.0150	0.2954 ± 0.1049	0.3504 ± 0.1104	0.0060
trans_s_s	0.1263 ± 0.1764	0.2052 ± 0.1868	0.0190	0.1379 ± 0.1895	0.2161 ± 0.1893	0.0273
trans_e_e	0.1616 ± 0.2136	0.0775 ± 0.1687	0.0254	0.1199 ± 0.1980	0.0495 ± 0.1167	0.0335
max_run_p	5.052 ± 3.689	6.810 ± 5.214	0.0255	8.594 ± 4.383	10.571 ± 4.733	0.0187
trans_p_p	0.3435 ± 0.1786	0.4165 ± 0.1800	0.0293	0.5069 ± 0.1145	0.5473 ± 0.0971	0.0483
freq_s	0.0181 ± 0.0240	0.0278 ± 0.0255	0.0333	0.0343 ± 0.0328	0.0513 ± 0.0393	0.0096
time_since_last_h	30.713 ± 355.326	–94.849 ± 267.687	0.0425	–12.554 ± 329.848	–129.851 ± 348.545	0.0610
dur_std_k	1.2926 ± 0.8803	1.7014 ± 1.4653	0.0446	3.5122 ± 2.8926	4.6971 ± 4.0899	0.0543
dur_mean_k	2.0772 ± 0.7251	2.3796 ± 1.0266	0.0503	7.8145 ± 4.7732	10.6927 ± 8.9094	0.0150
trans_h_k	0.1460 ± 0.0947	0.1138 ± 0.0784	0.0552	0.1563 ± 0.1611	0.1116 ± 0.1268	0.1138
2gram_m_p	0.0349 ± 0.0263	0.0446 ± 0.0287	0.0555	0.0217 ± 0.0116	0.0270 ± 0.0156	0.0291
dur_sum_s	14.3056 ± 30.7969	25.4443 ± 33.9446	0.0603	34.6293 ± 45.0479	52.1424 ± 43.6945	0.0358
2gram_p_m	0.0356 ± 0.0266	0.0445 ± 0.0269	0.0754	0.0371 ± 0.0148	0.0436 ± 0.0145	0.0184
dwell_before_std_s	78.2188 ± 145.3837	124.9827 ± 139.9487	0.0810	75.3926 ± 103.7871	120.2381 ± 171.9135	0.0608
duration_sum	846.0862 ± 590.8864	1033.8804 ± 598.6577	0.0893	952.2324 ± 648.7883	1162.9625 ± 748.5010	0.0964
trans_p_g	0.1460 ± 0.1579	0.1010 ± 0.0990	0.0907	0.0361 ± 0.0489	0.0197 ± 0.0262	0.0429
dur_sum_m	251.6336 ± 232.3093	324.8925 ± 241.8451	0.0946	115.4164 ± 77.2925	138.2658 ± 76.8012	0.1117
trans_m_p	0.1246 ± 0.0832	0.1507 ± 0.0847	0.0950	0.1588 ± 0.0761	0.1936 ± 0.0978	0.0253

Values reported as mean ± standard deviation
